# Short-term supplementation of thymol and carvacrol regulates feed intake via the hypothalamic PI3K/AKT/SF-1 pathway and influences intestinal traits in chicks

**DOI:** 10.3389/fvets.2026.1930378

**Published:** 2026-08-31

**Authors:** Ling Zhou, Zhiyong Huang, Jiancong Zhang, Xin Li, Shiping Bai, Fan Lei, Yutong Zou, Yue Zhang, Pinyao Zhao, Liguo Yin, Li Lv

**Affiliations:** 1 Solid-State Fermentation Resource Utilization Key Laboratory of Sichuan Province, Faculty of Quality Management and Inspection Quarantine, Sichuan Higher Education Engineering Research Center for Agri-Food Standardization and Inspection, Yibin University, Yibin, China; 2 Key Laboratory of Animal Disease-Resistance Nutrition, Ministry of Agriculture and Rural Affairs, Key Laboratory of Sichuan Province, Animal Nutrition Institute, Sichuan Agricultural University, Chengdu, China; 3 Institute of Brain Science and Diseases, Frontiers Science Center for Disease-related Molecular Network, West China Hospital, Sichuan University, Chengdu, Sichuan, China

**Keywords:** feed intake, intestinal traits, PI3K/AKT/SF-1 pathway, short-term feeding, thymol and carvacrol

## Abstract

**Introduction:**

This study investigated the effects of dietary supplementation with thymol, carvacrol, and NE150 (a commercial blend containing 25% thymol and 25% carvacrol) at 120 mg/kg on growth performance and feed intake, hypothalamic appetite-regulating gene expression and PI3K/AKT/SF-1 signaling, as well as intestinal morphology and cecal microbiota in broiler chicks.

**Methods:**

Based on preliminary dose-finding experiments (60, 120, and 240 mg/kg), the 120 mg/kg dose was selected for each additive as it showed the most favorable effects on growth performance (data not shown). A total of 180 one-day-old Arbor Acres male chicks were fed a basal diet from day 1 to 10, followed by a 3-day treatment period (days 10-13) with the respective diets.

**Results:**

Compared with the control group, total feed intake at 72 h was reduced by thymol, carvacrol, and NE150 (*P* < 0.05). Dietary supplementation with these compounds at 120 mg/kg upregulated the hypothalamic mRNA expression of pro‑opiomelanocortin and steroidogenic factor-1 (SF-1), while downregulating neuropeptide Y (*P* < 0.05). These treatments also increased the mRNA levels of cholecystokinin and glucagon‑like peptide‑1 receptor in both the hypothalamus and jejunum, and enhanced the protein expression of SF-1, p-PI3K, p-AKT473, p-AKT308, p-S6, and p-mTOR in the hypothalamus (*P* < 0.05). Furthermore, supplementation with carvacrol or NE150 improved the jejunal villus height to crypt depth ratio, and all three additives modulated the cecal microbiota by increasing microbial richness and diversity, as indicated by higher OTU numbers and Shannon index (*P* < 0.05).

**Discussion:**

These findings indicate that thymol and carvacrol suppress feed intake in broiler chicks, at least in part, through activation of the hypothalamic PI3K/AKT/SF-1 pathway, while also improving intestinal morphology and cecal microbiota composition. These results suggest that thymol and carvacrol may serve as effective antibiotic alternatives in poultry nutrition.

## Introduction

The misuse of antibiotics as growth promoters in chick feed has raised significant concerns regarding the transfer of antibiotic resistance genes from animals to human microbiota and the consequent development of antimicrobial resistance (1). In response, several countries have banned antibiotic additives in poultry diets, increasing interest in natural alternatives. Plant extracts are now explored as substitutes to enhance productivity and ensure the safety of poultry products (2, 3).

Thymol and carvacrol, the primary constituents of thyme and oregano essential oils, are used as feed additives in poultry nutrition due to their antioxidative, antimicrobial, and antiviral properties (4). Numerous studies indicate that these compounds, whether administered individually or in combination, can improve performance parameters and feed utilization efficiency, particularly by modulating digestion, metabolism, and gut microbiota (5–8). However, their effects on feed intake (FI) appear inconsistent. For instance, Hashemipour reported that dietary thymol reduced FI in poultry during days 10–20 but had no effect during days 0–10 (9). This suggests that the impact of carvacrol and thymol may vary with the feeding phase (10), yet research on the underlying mechanisms during short-term feeding remains limited.

The short-term regulation of feed intake in mammals and birds is primarily governed by peripheral satiation signals that control meal initiation and termination on a meal-to-meal basis, typically within 30 min to 5 h (11). These signals include mechanical, chemical, and hormonal cues from the gut, such as cholecystokinin (CCK), peptide YY (PYY), bombesin, and ghrelin, which are transmitted via the vagus nerve and brainstem to the hypothalamus, where they modulate the activity of orexigenic (NPY/AgRP) and anorexigenic (POMC) neurons (12, 13). Notably, ghrelin exhibits species-specific effects: it stimulates feeding in mammals but suppresses feed intake in chickens. Importantly, these gut brain signals are short lived and therefore play a key role in satiation and meal size control, and their disruption can lead to eating disorders and body weight changes (14). In broilers, the first 14 days post-hatch represent a critical period of rapid intestinal development and functional maturation, during which the gut is particularly responsive to dietary interventions (15). Therefore, to specifically evaluate the acute effects of thymol and carvacrol on feeding behavior and intestinal physiology during this sensitive developmental window, the present study restricted dietary supplementation to days 10–13 of age.

The arcuate nucleus (ARC) of the hypothalamus is a central regulator of feeding behavior, housing antagonistic orexigenic (NPY/AgRP) and anorexigenic (POMC) neurons that respond to metabolic hormones such as insulin and leptin (16). In chickens, conserved homologs of these neuropeptides form a hypothalamic melanocortin system sensitive to energy changes (17–19). Intracellularly, the phosphatidylinositol-3-kinase/protein kinase B (PI3K/Akt) signaling pathway plays a crucial role: central insulin administration elevates hypothalamic phosphorylated Akt (p-Akt) and reduces FI in poultry (20). Moreover, the PI3K/Akt pathway enhances the transcriptional activity of steroidogenic factor-1 (SF-1), a key regulator of feeding and energy homeostasis (21, 22). SF-1 is expressed in the poultry hypothalamus (23), and its neuronal activation rapidly suppresses FI in mice (24–26).

Although the central appetite-regulating pathways are well characterized, whether thymol and carvacrol influence these pathways in chickens remains unknown. Notably, thymol and carvacrol have been reported to modulate the PI3K/Akt pathway in non-poultry models: thymol protects against pulmonary fibrosis and carvacrol alleviates aortic hypercontractility in mice via this pathway (27, 28). However, whether thymol and carvacrol regulate FI through the hypothalamic PI3K/AKT/SF-1 pathway in chickens has never been examined. Furthermore, these compounds significantly shape the intestinal microbiota of poultry and exert pronounced physiological effects during the critical 10–20-day post-hatch period of intestinal development (29). To date, no study has investigated how short-term thymol or carvacrol treatment affects intestinal morphology and cecal microflora in chicks. Therefore, the present study aimed to address these gaps by evaluating the effects of dietary supplementation with thymol (120 mg/kg), carvacrol (120 mg/kg), and NE150 (a 1:1 blend of thymol and carvacrol, providing a total of 120 mg/kg of active compounds) on performance, the hypothalamic PI3K/AKT/SF-1 pathway, appetite-regulating gene expression, intestinal morphology, and cecal microflora in male Arbor Acres broiler chicks, compared with an unsupplemented control group.

## Materials and methods

### Ethics statement

All experimental procedures involving animals in this study were conducted according to the Animal Welfare Committee guidelines and received approval from the Animal Care and Use Committee of Sichuan Agricultural University (approval number: 20220052). Animal experiments adhered to the ARRIVE guidelines.

### Experimental design and chick management

Based on preliminary experiments, 120 mg/kg was identified as the optimal dose for each additive, which improved growth performance compared to other doses (data not shown). A total of 180 one-day-old male Arbor Acres chicks were weighed and randomly allocated to 4 treatment groups (45 chicks per group) and fed a basal diet from days 1 to 10. At the end of day 10, the chicks were fasted for 8 h, weighed, and then re-allocated to ensure that there were no significant differences in body weight among the treatment groups. From days 10 to 13, chicks were housed individually and provided with diets containing thymol (120 mg/kg; Sigma-Aldrich, Shanghai, China), carvacrol (120 mg/kg; Sigma-Aldrich, Shanghai, China), or NE150 (120 mg/kg; containing 25% thymol and 25% carvacrol as active components; Phibro Animal Health Co., Ltd., Shanghai, China), with a control group receiving no supplementation. All diets were pelleted and formulated to meet Arbor Acres chicken nutritional standards (Table 1). Throughout the 13-day experiment, chicks were managed under Arbor Acres-recommended conditions with ad libitum access to feed and antibiotic-free water. Room temperature was maintained at 35 °C during the first week and then gradually reduced by 2–3 °C weekly to a final 32 °C, with ambient humidity kept at 70%.

**Table 1:** Composition and nutrient levels of basal diets (air-dry basis).

Ingredients	Contents, %	Calculated nutrient levels	Contents
Corn	52.56	AME (kcal/kg)	3,000
Soybean meal (46%)	39.01	Crude protein %	21.50
Soybean oil	4.29	Calcium %	1.00
Calcium carbonate	1.00	Available phosphorus %	0.45
Calcium hydrogen phosphate	2.05	Lysine %	1.15
Sodium chloride	0.40	Methionine %	0.50
DL-Methionine	0.27	Tryptophan %	0.21
Vitamin premix^1^	0.05	Methionine + cysteine %	0.91
Mineral premix^2^	0.20	Threonine %	0.72
Choline chloride	0.17		

Vitamins^1^ and minerals^2^ were provide the followings per kilogram of diet: vitamin A, 8,000 IU; vitamin D3, 1,600 IU; vitamin E, 5 IU; vitamin K3, 4.0 mg; vitamin B1, 0.8 mg; vitamin B2, 2.50 mg; vitamin B6, 1.50 mg; vitamin B12, 1.50 mg; folic acid, 0.25 mg; niacin, 20 mg; pantothen, 2.20 mg; biotin, 0.10 mg; Fe (FeSO4·H2O), 60.0 mg; Cu (CuSO4·5H2O), 8.0 mg; Mn (MnSO4·H2O), 60.0 mg; Zn (ZnSO4·H2O), 80.0 mg; Se (Na2SeO3), 0.30 mg; I (KI), 0.35 mg.

### Growth performance

Total FI and residual feed for each replicate in individual cages were recorded at 12 h, 24 h, and 72 h after providing the treatment diets. Body weight was recorded at the end of day 10 and again at the end of day 13 to calculate body weight gain during the treatment period. Feed conversion ratio (FCR) was calculated as total FI (72 h cumulative intake) divided by body weight gain over the 3-day period (days 10–13).

### Sample collection

On day 13, eight chicks from each treatment were euthanized. Hypothalamus, jejunal and cecal contents were collected. Hypothalamus and cecal contents samples were flash frozen in liquid nitrogen and stored at −80 °C. For histology, 2-cm segments of jejunum were fixed in 4% paraformaldehyde.

### Total RNA extraction, cDNA synthesis, and real-time PCR

Total RNA was extracted from hypothalamus using TRIzol™, quantified by NanoDrop™ (OD260/280 = 1.8–2.0), and reverse-transcribed with PrimeScript™ RT kit. qPCR was performed on a QuantStudio5® system using SYBR Green (primers in Table 2). Reactions were run in triplicate, with *β*-actin as internal control. Relative expression was calculated by 2^−ΔΔCT^ method.

**Table 2:** Sequences of primers and probes.

Genes	Orientation	Primer sequence (5′ → 3′)	Gene number
*SF1*	Forward	ATGGACTATTCGTATGATGAGG	NM_205077
	Reverse	AACTGCTGGGGTAGGTCTCT	
*POMC*	Forward	GGAGAACAGCAAGTGCCAGGAC	NM_001031098
	Reverse	CACACGCCAAAACACCAGCC	
*CCK*	Forward	GATGGCAGCTTCGAGCAGAG	NM_001001741
	Reverse	GTCATTTATCCTGTGTGGGATC	
GLP1R	Forward	CCCATGAGGTCATCTTTGCC	NM_001135551.1
	Reverse	TGCCTCCACTACTGATGCTG	
*NPY*	Forward	AGAAGCGTACCCCTCCAAAC	NM_205473
	Reverse	ACCACATCGAAGGGTCTTCA	
*β-Actin*	Forward	TGCGTGACATCAAGGAGAAG	NM_205518
	Reverse	TGCCAGGGTACATTGTGGTA	

SF1, steroidogenic factor-1; POMC, proopiomelanocortin; CCK, cholecystokinin; GLP1R, glucagon-like-peptide-1 incretin receptor; NPY, neuropeptide Y.

### Western blot analysis

Hypothalamic proteins were extracted in lysis buffer with protease/phosphatase inhibitors, quantified by BCA assay, separated by 10% SDS-PAGE, and transferred to PVDF membranes. After blocking, membranes were incubated with primary antibodies (PI3K, p-PI3K, Akt, p-Akt308, p-Akt473, mTOR, p-mTOR, S6, p-S6, *β*-actin; Supplementary Table S1) overnight at 4 °C, then with HRP-conjugated secondary antibodies. Bands were visualized using enhanced chemiluminescence and quantified with ImageJ.

### 16S microbial detection and analysis

Cecal contents DNA was extracted and submitted to Beijing Nohe Biotechnology Co., Ltd. for 16S rDNA amplicon sequencing. Operational taxonomic units (OTUs) were clustered, and microbial composition was analyzed at phylum to genus levels. Alpha diversity (Shannon, Simpson) and beta diversity (weighted UniFrac PCoA) were evaluated.

### Histological analysis

Fixed jejunum samples were dehydrated, embedded in paraffin, sectioned (5 μm), and stained with hematoxylin and eosin. Villus height (VH) and crypt depth (CD) were measured from 10 randomly selected villus-crypt units per section using Image-Pro Plus 6.0, and the VH/CD ratio was calculated.

### Statistical analyses

The experiment employed a completely randomized design. Each pen served as the experimental unit for production performance data (*n* = 45), whereas individual birds were used for other physiological indicators (*n* = 8 per treatment). Data were analyzed by one-way ANOVA using SPSS (version 26.0; IBM SPSS Statistics, Armonk, NY, United States). Duncan’s multiple-range test was used for post-hoc comparisons, with statistical significance set at *p* < 0.05. Results are reported as the mean ± SEM. Significance levels are denoted as follows: *p < 0.05, **p < 0.01, and ***p < 0.001.

## Results

### Growth performance

Compared with the control group, total FI at 72 h was lower in the thymol, carvacrol, and NE150 groups (*p* < 0.05; Table 3). Body weight gain (11–13 d) and final body weight (13 d) showed numerical increases across all treatment groups, although no statistically significant differences were detected (*p* > 0.05; Table 3). Similarly, FCR was numerically improved in all supplemented groups, but the differences were not significant compared with the control group (*p* > 0.05; Table 3).

**Table 3:** Effects of plant essential oils on performance of chick (*n* = 45).

Item	Supplemental level (120 mg/kg)				SEM	*p*-value
	Control	Thymol	Carvacrol	NE150		
Feed intake/g						
12 h	27.81	26.08	26.11	25.81	0.65	0.70
24 h	47.45	44.68	45.96	44.32	0.88	0.59
72 h	177.67^a^	172.09^b^	171.76^b^	169.92^b^	0.99	0.03
Body weight gain/g (11d–13d)	160.43	175.27	170.64	169.13	5.14	0.78
Body weight/g (13d)	476.62	492.04	487.63	486.03	4.53	0.68
FCR (11d–13d)	1.14	1.04	1.07	1.10	0.034	0.79

^a,b^Means within the same row having different superscripts differ significantly (*P* < 0.05). NE150: containing 25% thymol and 25% carvacrol as active components; FCR, feed conversion ratio.

### Expression of hypothalamic feeding-related genes

**Figure 1:**
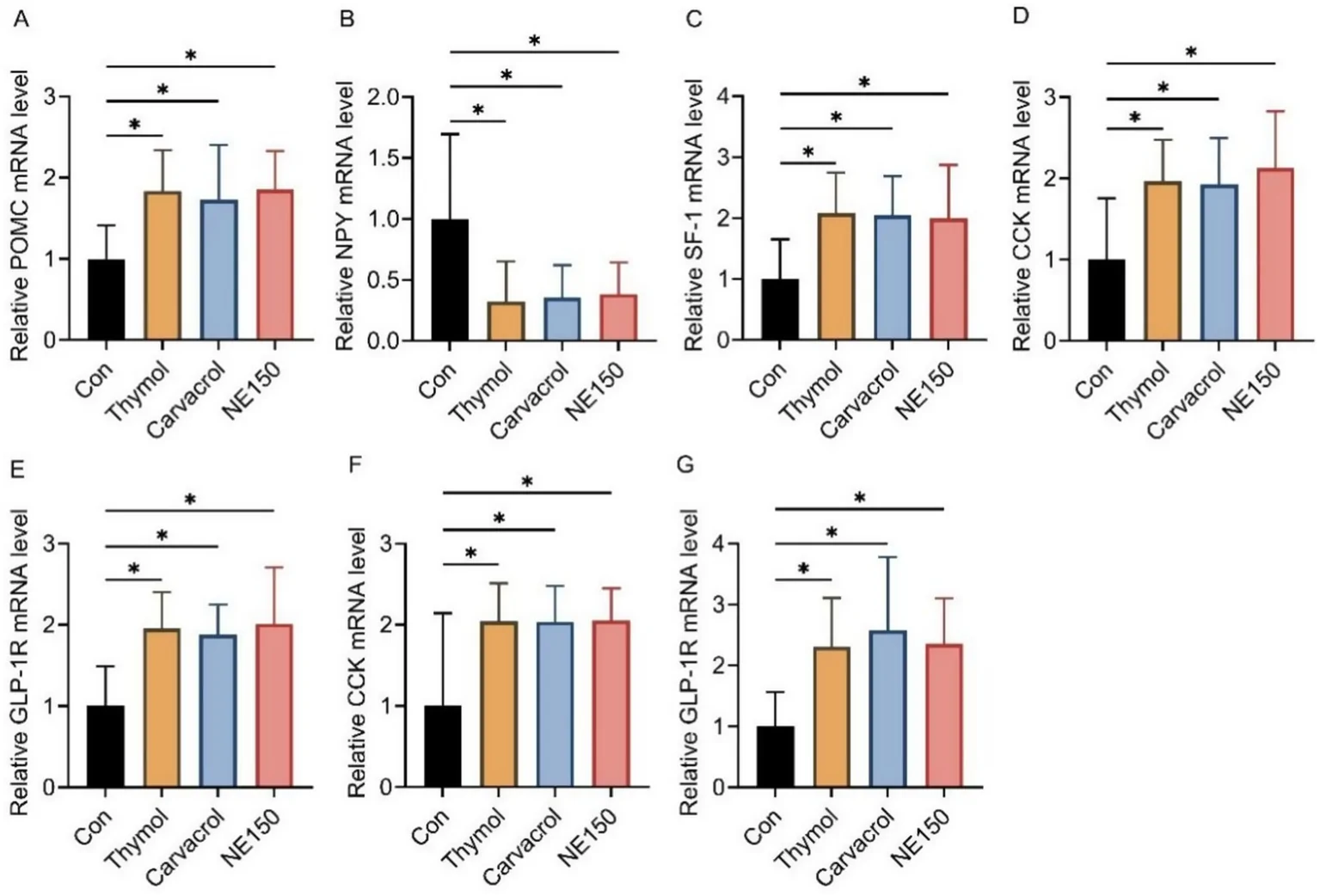
Effects of 120 mg/kg of thymol, carvacrol, and NE150 on feeding-related genes in the hypothalamus and jejunum (*n* = 8). **(A–E)** mRNA expression level in the hypothalamus. **(F,G)** mRNA expression level in the jejunum. POMC, proopiomelanocortin; NPY, neuropeptide Y; SF-1, steroidogenic factor-1; CCK, cholecystokinin; GLP-1R, glucagon-like-peptide-1 incretin receptor. NE150: Containing 25% thymol and 25% carvacrol as active components group. * *p* < 0.05.

As illustrated in Figure 1, the hypothalamic mRNA expression of *POMC*, *SF-1*, *CCK*, and glucagon-like peptide-1 receptor (*GLP-1R*) was upregulated (*p* < 0.05), whereas that of *NPY* was downregulated in response to the dietary inclusion of thymol, carvacrol, and NE150 over the 3-day treatment period compared with that in the control group. Moreover, the mRNA expression levels of *CCK* and *GLP-1R* in the hypothalamus and jejunum were significantly elevated.

### Expression of the hypothalamic PI3K/AKT/SF-1 pathway

**Figure 2:**
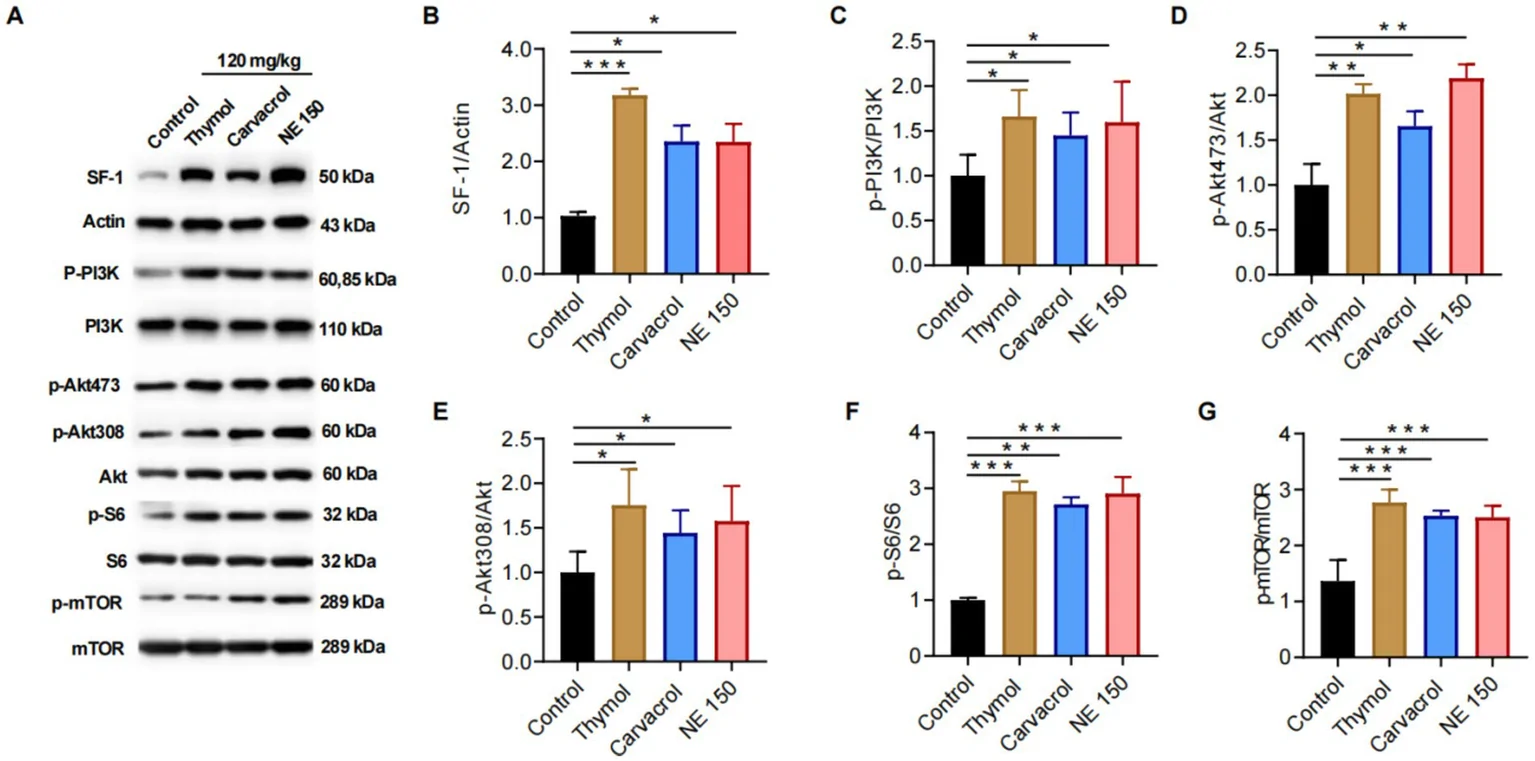
Effects of 120 mg/kg of thymol, carvacrol, and NE150 on PI3K/AKT signaling pathway in hypothalamus (*n* = 8). **(A)** Representative immunoblots of all protein. The protein expressions of **(B)** SF-1; **(C)** p-PI3K; **(D)** p-AKT473; **(E)** p-AKT308; **(F)** p-S6; **(G)** p-mTOR expression level. NE150: Containing 25% thymol and 25% carvacrol as active components group. * *p* < 0.05, ** *p* < 0.01, and *** *p* < 0.001.

To investigate the involvement of the PI3K/AKT/SF-1 pathway in the reduced FI associated with thymol, carvacrol, and NE150, protein expression related to this pathway was analyzed via Western blotting. As depicted in Figure 2, elevated protein levels of p-PI3K, p-AKT308, p-AKT473, SF-1, p-mTOR, and p-S6 were observed in the thymol, carvacrol, and NE150 groups compared with those in the control group (*p* < 0.05).

### Intestinal morphology

**Figure 3:**
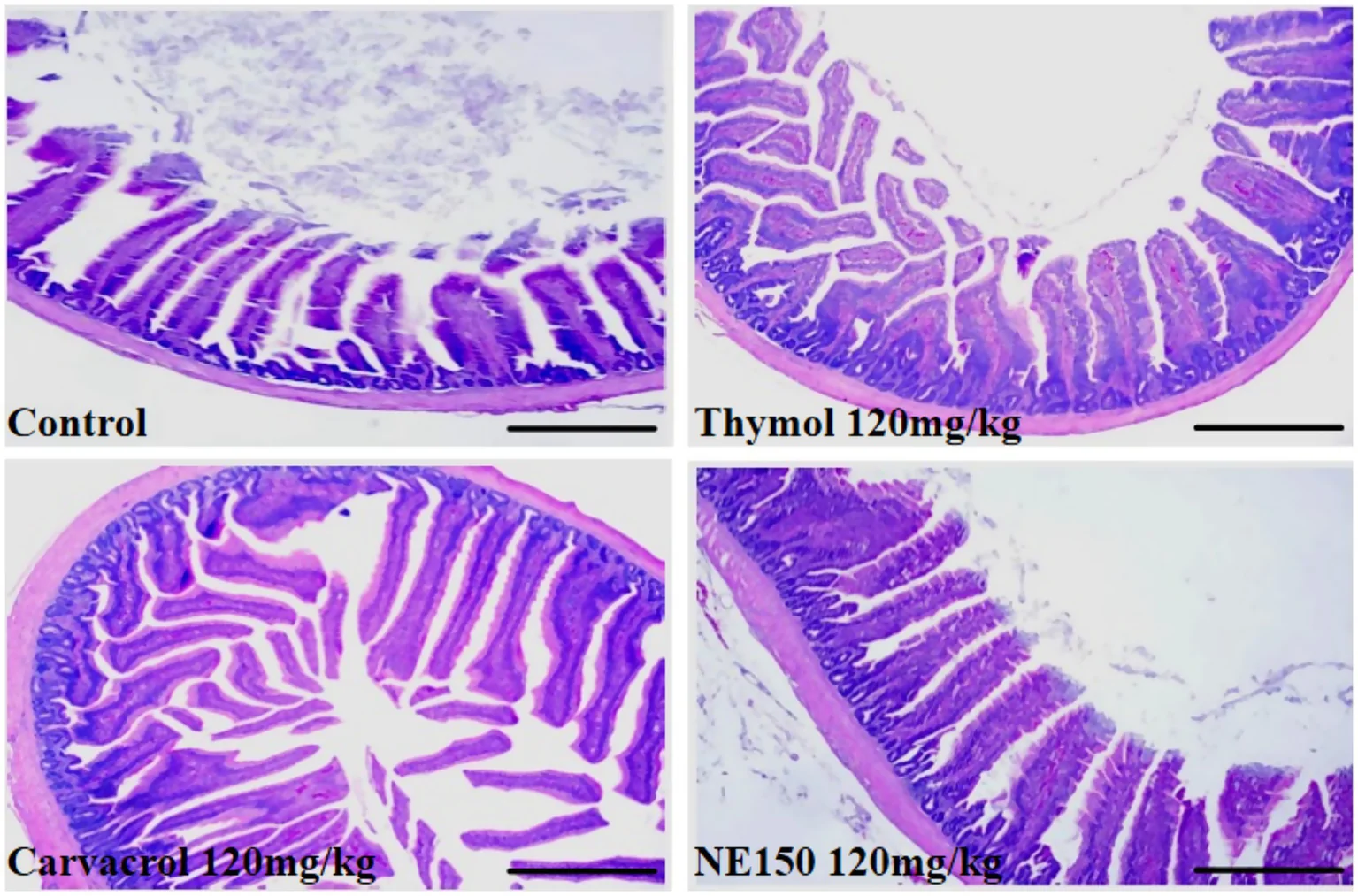
Representative histological images of intestinal tissue from 13-day-old chickens in different experimental groups (*n* = 8). Bar = 100 μm. NE: Containing 25% thymol and 25% carvacrol as active components group.

Table 4 indicates that the various dietary groups exerted no significant effects on VH and CD in the jejunum (*p* > 0.05). Notably, the carvacrol-supplemented group exhibited the highest VH, measuring 780.75 μm. Throughout the 3-day treatment period, the ratio of jejunal VH to CD increased in both the carvacrol and NE150 groups (*p* < 0.05). Although not statistically significant, the thymol-supplemented group also demonstrated an upward trend in this ratio (Figure 3).

**Table 4:** Effects of 120 mg/kg of thymol, carvacrol and NE150 on intestinal morphology (*n* = 8).

Item	Supplemental level (120 mg/kg)				SEM	*P*-value
	Control	Thymol	Carvacrol	NE150		
Villus height/μm	763.56	766.65	780.75	773.78	6.06	0.51
Crypt depth/μm	96.91	100.80	100.80	105.05	0.88	0.28
VH:CD ratio	7.84^c^	8.22^bc^	9.39^a^	8.64^b^	0.72	0.03

^a,b,c^Means within the same row having different superscripts differ significantly (*P* < 0.05). VH, villus height; CD, crypt depth. NE150: containing 25% thymol and 25% carvacrol as active components group.

### Structure of cecal bacterial community

**Figure 4:**
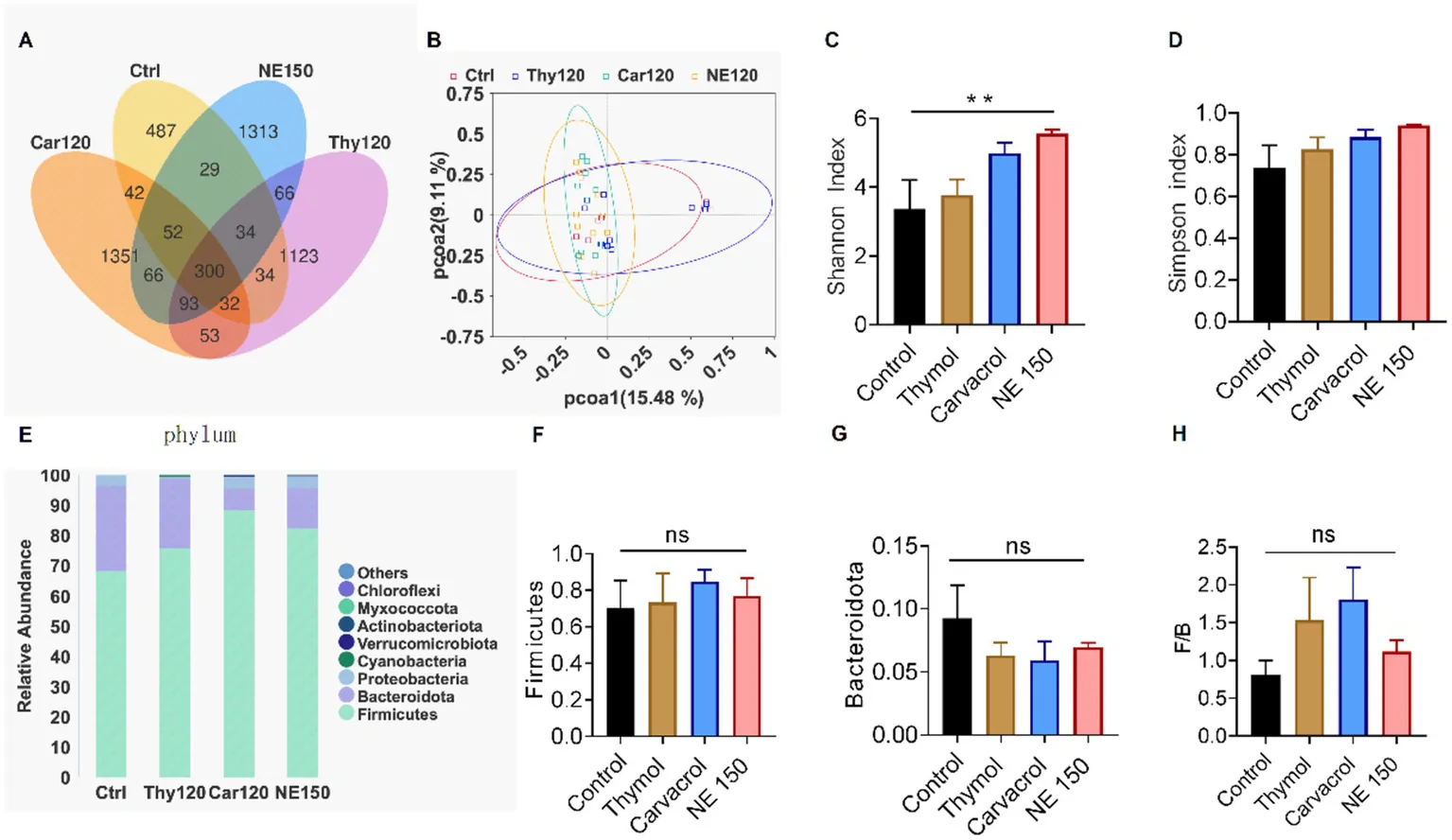
Effects of 120 mg/kg of thymol, carvacrol, and NE150 on the intestinal microbiota and related parameters in the experimental groups (*n* = 8). **(A)** Venn diagram of OTUs in different groups; **(B)** P-CoA plot of cecal microbiota beta-diversity; **(C,D)** Shannon and Simpson indices for cecal microbiota alpha-diversity; **(E)** Relative abundances of major cecal microbiota phyla; **(F–H)** Relative abundances of Firmicutes, Bacteroidetes, and Firmicutes/Bacteroidetes ratio in cecal microbiota. Con: Control group; Thy120: 120 mg/kg thymol group; Car120: 120 mg/kg thymol group; NE150: Containing 25% thymol and 25% carvacrol as active components group. Ns: Not significant. * *p* < 0.05, ** *p* < 0.01.

Cecal microbial composition was assessed by 16S rRNA sequencing. As shown in Figure 4, all groups shared 300 common OTUs, while the control, thymol, carvacrol, and NE150 groups exhibited 487, 1,123, 1,351, and 1,313 unique OTUs, respectively. The Shannon index was higher in the NE150 group than in the control group (*p* < 0.05), indicating increased microbial diversity. At the phylum level, Firmicutes, Bacteroidetes, and Proteobacteria accounted for 95–99% of all sequences. Dietary supplementation with thymol, carvacrol, and NE150 showed no significant effects on the relative abundance of Firmicutes or Bacteroidetes, nor on the Firmicutes/Bacteroidetes ratio (*p* > 0.05).

## Discussion

The present study discovered that dietary supplementation with thymol, carvacrol, and NE150 at 120 mg/kg positively affected growth performance in broiler chickens. Furthermore, thymol and carvacrol evidently reduced FI in chickens. This finding aligns with previous literature; for instance, one investigation noted that carvacrol reduced FI, weight gain, and the FCR when chicks were fed a diet supplemented with 200 mg/kg carvacrol (30). Cross reported an increase in BW gain in chicks supplemented with thyme essential oil at a level of 1,000 mg/kg, despite a nearly 10% reduction in FI (31). In the present study, total FI at 72 h was significantly reduced by thymol, carvacrol, and NE150 compared with the control group, accompanied by numerical improvements in final body weight and FCR at 13 days of age. These results suggest that thymol and carvacrol can reduce feed intake while maintaining or improving growth efficiency, which is consistent with the notion that these compounds enhance nutrient utilization rather than simply promoting greater consumption.

Additionally, thymol and carvacrol significantly influence FCR reduction by stabilizing the microbial population and enhancing nutrient absorption (8, 32). They also activate digestive enzymes, improving digestion. One study found that supplementation with thymol and carvacrol increased the activities of intestinal and pancreatic enzymes, including trypsin, lipase, and protease, in 24-day-old chicks (9). Moreover, supplementation with an oregano oil product containing carvacrol and thymol as active ingredients resulted in a quadratic increase in ileal chymotrypsin activity along with a linear and quadratic increase in ileal lipase activity in late-phase laying hens (33). Additionally, Lee found that carvacrol and thymol improved the FCR in chicks by inactivating insulin sites in the liver, leading to enhanced feed utilization efficiency (30). In the current study, the numerical improvement in FCR observed in the supplemented groups may be attributable to similar mechanisms, including enhanced digestive enzyme activities and improved nutrient absorption, which compensated for the reduced feed intake.

Research indicates that both thymol and carvacrol readily cross the blood–brain barrier, providing neuroprotective effects (34). FI is partially regulated by neuropeptides expressed in neurons located in the nuclei of the mediobasal hypothalamus. The hypothalamic ARC region contains first-order neurons that govern FI and energy balance: orexigenic neuropeptides (e.g., NPY and AgRP) stimulate FI, while *α*-melanocyte-stimulating hormone, derived from POMC, serves as an anorexigenic neuropeptide that moderates FI (34, 35). Notably, this study found that feeding chickens thymol, carvacrol, and NE150 resulted in a pronounced reduction in the expression of orexigenic hormones like NPY and an increase in the mRNA expression levels of the anorexigenic peptide POMC in the hypothalamus. These molecular changes are consistent with the observed reduction in feed intake, suggesting that thymol and carvacrol modulate feeding behavior at least in part through the hypothalamic melanocortin system.

In a related study, Yang et al. (34) observed minimal changes in FI following the injection of wortmannin, a PI3K inhibitor, prior to exendin-4 (EX-4) intraperitoneal injection. Zhao et al. (36) utilized male Sprague–Dawley rats implanted with intracerebroventricular (ICV) cannulae and demonstrated that the ICV administration of leptin elevated hypothalamic PI3K levels while simultaneously reducing FI. As previously noted, PI3K plays a vital role in regulating FI. A key downstream target of PI3K is protein kinase B/Akt, which is activated via serine and threonine phosphorylation. Fujita et al. (37) research reveals that ICV administration of insulin-like growth factor I in poultry significantly enhances the hypothalamic expression of p-AKT and concurrently diminishes FI. This study found that in chickens, thymol, carvacrol, and NE150 increase the expression of p-PI3K, p-AKT, SF-1, p-mTOR, and p-S6 in the hypothalamus. These results suggest that thymol and carvacrol inhibit FI via the PI3K/AKT/SF-1 signaling pathway in the hypothalamus, providing a mechanistic link between dietary supplementation and central appetite regulation.

CCK is a brain–gut peptide that serves a crucial role in inhibiting FI and promoting digestive and absorptive functions (38). Chen et al. (39) reported that the injection of CCK into the dorsomedial hypothalamus of male Sprague–Dawley rats resulted in a rapid decrease in FI, inhibiting consumption for 4 h without compensation for at least the subsequent 18 h. This intervention also led to decreased NPY gene expression in the dorsomedial hypothalamus (39). Furthermore, a study indicated that the ICV injection of CCK8s (1 nmol) in birds significantly reduced FI compared with the control (40). Additionally, CCK stimulates the secretion of pancreatic juice, which is rich in digestive enzymes, and promotes gallbladder contraction to release bile, both of which facilitate the digestion of fats and other nutrients (41). Consistent with this, thymol, carvacrol, and NE150 significantly elevated jejunal and hypothalamic CCK mRNA levels, correlating with the anorexic effects of CCK. The concurrent elevation of CCK in both the hypothalamus and jejunum suggests that thymol and carvacrol may activate the brain–gut axis to coordinate feeding inhibition and digestive preparation.

GLP-1 plays a significant role in regulating FI and energy metabolism (42). In 1996, Turton et al. (43) first reported that the ICV administration of GLP-1 suppresses FI in mammals. Consequently, GLP-1 is recognized as an anorexigenic neurotransmitter in these species. In chickens, GLP-1R is highly expressed in the telencephalon, midbrain, hindbrain, and hypothalamus (44). Furuse demonstrated that the ICV administration of chicken GLP-1 significantly suppressed FI in chickens (45). A previous study suggested that the GLP-1R agonist EX-4 reduced FI and BW by altering POMC mRNA expression in the hypothalamus of rats (46). Similarly, this article reveals that GLP-1R mRNA levels were significantly elevated by thymol, carvacrol, and NE150 in both the hypothalamus and jejunum. This upregulation of GLP-1R expression may potentiate the anorexigenic action of endogenous GLP-1, thereby contributing to the reduced feed intake observed in the supplemented groups.

Healthy intestinal morphology is essential for optimal growth performance. Increased VH and decreased CD enhance nutrient absorption by providing a greater surface area and facilitating a faster rate of nutrient absorption (47). This study demonstrated a significant increase in the jejunal VH/CD ratio in the carvacrol and NE150 groups compared with the control group. An increase in this ratio enhances nutrient absorption in the small intestine, thereby influencing chicken production. In a previous study, Michiels et al. (4) indicated that supplementation with 500 ppm carvacrol and thymol reduces the number of intraepithelial lymphocytes while increasing the VH/CD ratio in the distal small intestine. The improved intestinal morphology observed in the present study likely contributed to the numerical improvements in body weight and FCR, as a healthier intestinal epithelium facilitates more efficient nutrient absorption even under conditions of reduced feed intake.

The complex communities of intestinal microbiota residing in the gut are essential for intestinal nutrient absorption, digestion, immune regulation, and overall intestinal health in the host (48, 49). Research has demonstrated that carvacrol and thymol possess antimicrobial properties beneficial for poultry production (50). Sidiropoulou observed no significant effect of Colombian oregano oil, chiefly comprising 0.9% carvacrol and 78.7% thymol, on cecal phyla and genera in coccidia-challenged chicks; however, a positive correlation was identified between BW and the Firmicutes/Bacteroidetes ratio (51). In the current study, all three additives modulated the cecal microbiota by increasing microbial richness and diversity, as reflected by higher OTU numbers and Shannon index. Although no significant changes were observed in the Firmicutes/Bacteroidetes ratio, the increased microbial diversity may still represent a favorable shift in gut health, consistent with the known antimicrobial properties of thymol and carvacrol.

In conclusion, the present study demonstrates that short-term dietary supplementation with thymol, carvacrol, and NE150 at 120 mg/kg reduces feed intake in broiler chicks through activation of the hypothalamic PI3K/AKT/SF-1 pathway and modulation of appetite-regulating neuropeptides and gut hormones gene expression. These central effects are accompanied by improvements in intestinal morphology and cecal microbiota composition, which likely contribute to the numerical improvements in growth performance despite reduced feed consumption. These findings provide new insights into the mechanisms by which phytogenic compounds regulate feed intake and growth in broilers, supporting their potential use as antibiotic alternatives in broiler production.

## Conclusion

Chick feed intake is influenced by short-term dietary inclusion of thymol, carvacrol, or NE150 at 120 mg/kg. This reduction in feed intake is attributed to the downregulation of orexigenic gene expression and the upregulation of anorexigenic gene expression in the hypothalamus. These alterations converge in the hypothalamus, where thymol, carvacrol, and NE150 stimulate the activation of the hypothalamic PI3K/AKT/SF-1 signaling pathway in chicks, accompanied by beneficial changes in intestinal morphology and microbiota.

## Data Availability

16S rRNA sequencing data have been deposited in the NCBI SRA under BioProject PRJNA1510894.
