# Supplementary material for: Short-term supplementation of thymol and carvacrol regulates feed intake via the hypothalamic PI3K/AKT/SF-1 pathway and influences intestinal traits in chicks
Source: Front Vet Sci. 2026 Aug 31;13:1930378. doi: 10.3389/fvets.2026.1930378 (PMC13572108; doi:10.3389/fvets.2026.1930378)
Supplement: Supplementary file 1 [file Table_1.docx]

**Table S1**. Primary antibodies for Western blot analysis

| **Name** | **Supplier** | **Cat no.** |
| --- | --- | --- |
| mTOR | Cell Signaling Technology, USA | 2983 |
| p-mTOR | Cell Signaling Technology, USA | 2971 |
| S6 | Cell Signaling Technology, USA | 2217 |
| p-S6 | Cell Signaling Technology, USA | 9234 |
| SF-1 | Santa Cruz Biotechnology, USA | sc-393592 |
| p-AMPK | Cell Signaling Technology, USA | 2531 |
| AKT | Cell Signaling Technology, USA | 9272 |
| p-AKT_473_ | Cell Signaling Technology, USA | 9271 |
| p-AKT_308_ | Cell Signaling Technology, USA | 9275 |
| PI3K | Cell Signaling Technology, USA | 4249 |
| p-PI3K | Cell Signaling Technology, USA | 4228 |
| β-Actin | Cell Signaling Technology, USA | 2148 |
| Anti-rabbit lgG | Cell Signaling Technology, USA | 7074 |
| Anti-mouse lgG | Cell Signaling Technology, USA | 7076 |
